# Simulating Time-Dependent Patterns of Nonadherence by Patients With Breast Cancer to Adjuvant Oral Endocrine Therapy

**DOI:** 10.1200/CCI.18.00091

**Published:** 2019-04-19

**Authors:** Eileen H. Shinn, Gordon Broderick, Bryan Fellman, Ainslee Johnson, Elizabeth Wieland, Stacy Moulder, William Fraser Symmans

**Affiliations:** ^1^ University of Texas MD Anderson Cancer Center, Houston, TX; ^2^ Rochester Institute of Technology, Rochester, NY

## Abstract

**PURPOSE:**

Nearly 40% of patients with breast cancer discontinue their adjuvant oral endocrine treatment (ET). We measured discontinuation rates of ET at a comprehensive cancer center. We then used an iterative approach to model patterns of determinants associated with discontinuation of ET.

**METHODS:**

Patients with nonmetastatic breast cancer receiving active adjuvant ET were approached by nurse practitioners to complete an anonymous survey at one time point. We simulated a prospective model by iteratively regressing adverse effects onto adherence status across windowed time periods of 2 to 3 consecutive years, bootstrapping the smaller group of nonadherent patients and subsampling the larger adherent group.

**RESULTS:**

From February to April 2013, 216 participants were enrolled in the study. Forty patients (18.5%) reported that they had discontinued ET during the first 5 years of ET, and an additional four patients (1.9%) missed > 20% of their doses. Using two-sided significance tests, simulations showed that all 13 ET adverse effects and reasons for discontinuation were significantly related to discontinuation at some time point during ET. Worry about ET cost (odds ratio [OR], 1.79), emotional distress (OR, 1.72), and bone and joint pain (OR, 1.69) were the three most impactful reasons for discontinuation, with varying patterns of influence over time.

**CONCLUSION:**

These analyses provide preliminary evidence that there are varying patterns of discontinuation of ET. Although some reasons for discontinuation exerted a steady influence over the 6-year ET trajectory (ie, bone and joint pain), other reasons, such as cost, cognitive complaints, and general dislike of pills, became more important in the later years of ET.

## INTRODUCTION

Despite the proven efficacy of adjuvant endocrine treatment (ET), early discontinuation of ET is a problem. By the end of the first year, patient discontinuation rates range from 7% to 14%; by the end of the fifth year, discontinuation rates range from 40% to 60%.^1-4^ Discontinuation of adjuvant ET during the first year is associated with increased risk of breast cancer–related mortality (hazard ratio [HR], 6.3) compared with patients who complete 5 years of ET.^5^ Patient reasons for discontinuation include painful adverse effects, forgetting, medication cost, and a limited sense of urgency within the context of lack of active disease.^3,6-8^ Demographic factors include racial minority status, having an educational background of high school degree or lower, and unemployment status.^9^

Patient nonadherence to chronic oral medication is a complex problem. The majority of extant medication adherence literature includes classical regression analyses, calculating the odds of becoming nonadherent given a participant’s profile of descriptive patient, symptom, or treatment features at baseline. However, in their basic form at least, classical regression models do not effectively capture the varying effects of predictor variables on outcome over time.^10^ Furthermore, data for these studies come primarily from secondary sources, such as insurance databases or clinical trials, the primary end point of which was disease-free survival.

We addressed these gaps in the literature in the following ways. First, we directly studied discontinuation of ET at a tertiary comprehensive cancer center in patients who were able to maintain their follow-up care at the MD Anderson (MDA) Breast Center. This cohort also offered an opportunity to study patient willingness to continue ET beyond 5 years, because beginning in 2005, patients with estrogen receptor (ER)–positive, human epidermal growth factor receptor (HER2)–negative early-stage breast cancer at MDA were offered extended adjuvant ET, after the initial trial results of MA.17.^11,12^ A secondary aim was to examine how patients’ reasons for discontinuation evolve over time. Given that our study design was correlational, we explored the simulation of a prospective approach by modeling our data across moving windows of time across the ET trajectory to maximize statistical power.

## METHODS

Patients who were attending routine surveillance visits were asked by their nurse practitioners to participate in an institutional review board–exempted anonymous survey; a consent statement was included. Patients who agreed completed the questionnaire and left it in a sealed envelope with their nurse practitioner. Neither the questionnaire nor the envelope had patient identifiers.

Patients were eligible if they had been previously treated for ER-positive stage I to III breast cancer and had been prescribed an antiestrogen hormonal treatment, were attending a surveillance visit at the MDA Breast Center, were ≥ 18 years of age, did not have recurrent disease, did not have a new cancer primary, and could read English well enough to complete the survey.

### Assessment of Adherence and Reasons for Discontinuation

The survey asked 20 questions, including the most recently prescribed ET, month and year of breast cancer diagnosis, current adherence status, total duration of adherence to ET, lymph node involvement, type of surgery and chemotherapy (if applicable), and demographic information (age, race, education, marital status).

Using a 5-point Likert response scale ranging from “not at all” to “very much,” participants were asked to rate the impact of 13 ET adverse effects and concerns on the decision to discontinue ET: worry about bone loss, bone and joint pain, low sex drive, vaginal dryness (pain during sex), hot flashes, worry about drug interaction with other existing medication, insomnia or trouble sleeping, weight gain attributed to ET, emotional distress attributed to ET, cognitive dysfunction, forgetting to take endocrine-blocking pill, cost of ET, and general dislike of taking medication.

### Assessment of Discontinuation and Nonadherence

If the patient indicated that she was still taking her daily ET at the time of the survey, duration of adherence to ET was calculated by subtracting the month and year the survey was answered from the month and year of breast cancer diagnosis. If the patient indicated that she had discontinued ET, the duration of adherence was calculated by subtracting the month and year that the patient reported discontinuing ET from the month and year of breast cancer diagnosis. If a participant indicated that she was still taking her daily ET but had missed doses > 20% of the time (and not at the direction of her physician), she was considered nonadherent.

### Statistical Analysis

Point prevalence adherence rates for each year of ET were calculated by dividing the number of patients prescribed hormonal therapy by the number who indicated on the questionnaire that they were still taking the medication. Demographic factors were also analyzed to determine relationship to adherence status.

### Simulating Nonadherence Over Time With Use of Windowed Time Periods

Logistic regression models predicting the probability of nonadherent status were constructed to quantify the contribution of each factor for nonadherence. Nonadherence was defined as either discontinuing ET or missing > 20% of doses. To account for the changing influence of the factors on nonadherence over time, separate models were repeated for each year of ET within a 2- to 3-year moving window as it shifted progressively in increments of 1 year. With the exception of the first and last time points of the ET trajectory, each windowed time period was centered at each year of ET and included the most immediate preceding and subsequent years. For example, the regression model for nonadherence during year 2 of ET was based on a subsample of participants who became nonadherent during the second year of ET, as well as participants who became nonadherent during the first and third years of ET. The next windowed time period was centered on year 3 of ET and included participants who had become nonadherent during the second, third, or fourth year of ET.

For the analysis centered on year 1, the number of patients who had become nonadherent during year 1 was relatively large, so year 1 was divided into three time periods: never initiated (Y0), became nonadherent during months 1 to 6, and became nonadherent during months 7 to 12. The first time period centered on those who had never initiated and those who became nonadherent during months 1 to 6; the next time period was centered on months 1 to 6 and included patients who never initiated and those who became nonadherent during months 7 to 12, and so on. We truncated the analysis of reasons for nonadherence at year 6, because the number of participants beyond the sixth year of ET was small (n = 10). For the analysis centered on year 6, the moving window included participants who had become nonadherent in years 5 and 6 only.

For each windowed time period, we calculated the median value of the logistic coefficient over 100 iterations on subsets that were balanced with the use of bootstrapping or subsampling with replacement using a uniform probability of selection. Median values were calculated for each adverse effect coefficient associated with discontinuation of ET within that time period. We then estimated the probability that changes in coefficients over time were random.

Classification bias resulting from differences in group size^13^ was avoided by repeating each regression on 100 subsamples within each windowed period. For each of the 100 iterations, the smaller group of nonadherent participants was bootstrapped, and the larger group of adherent participants was subsampled to create two groups (discontinued *v* adherent) of 30 participants each.^14^ Logistic regression was performed on each of these 100 subsets, and the scaled coefficient values, which expressed the effects of the reasons on the relative risk (RR) or log odds ratio (OR) of being in adherent versus nonadherent,^15^ were recorded to assess the stability of these estimates within each time window; median logistic coefficients for each time point of ET (those who had never initiated were designated as Y0) until year 6) and two-sided *P* values were calculated.

To calculate the probability that changes in coefficient medians across time periods represented a random sequence, we used a test based on the number of segments or runs of consecutive values above or below the mean expected value.^16^ Fewer runs than expected by chance indicate a significant change in impact over time, whereas more-than-expected runs indicate a random oscillation. All analyses were performed using the *bootstrp*, *mnrfit*, *ranova*, and *runstest* functions available in the MATLAB Statistics Toolbox (MathWorks, Natick, MA).

### Comparison of Severity Levels of Adverse Effects in Years 0 to 2 Versus 3 to 6

Likert-scale raw scores for each ET adverse effect or reason for discontinuation were summed and compared in adherent versus nonadherent groups in the early years of ET (never initiated [Y0], years 1 to 2) as well as the latter years of ET (years 3 to 6).

## RESULTS

**FIG 1.**
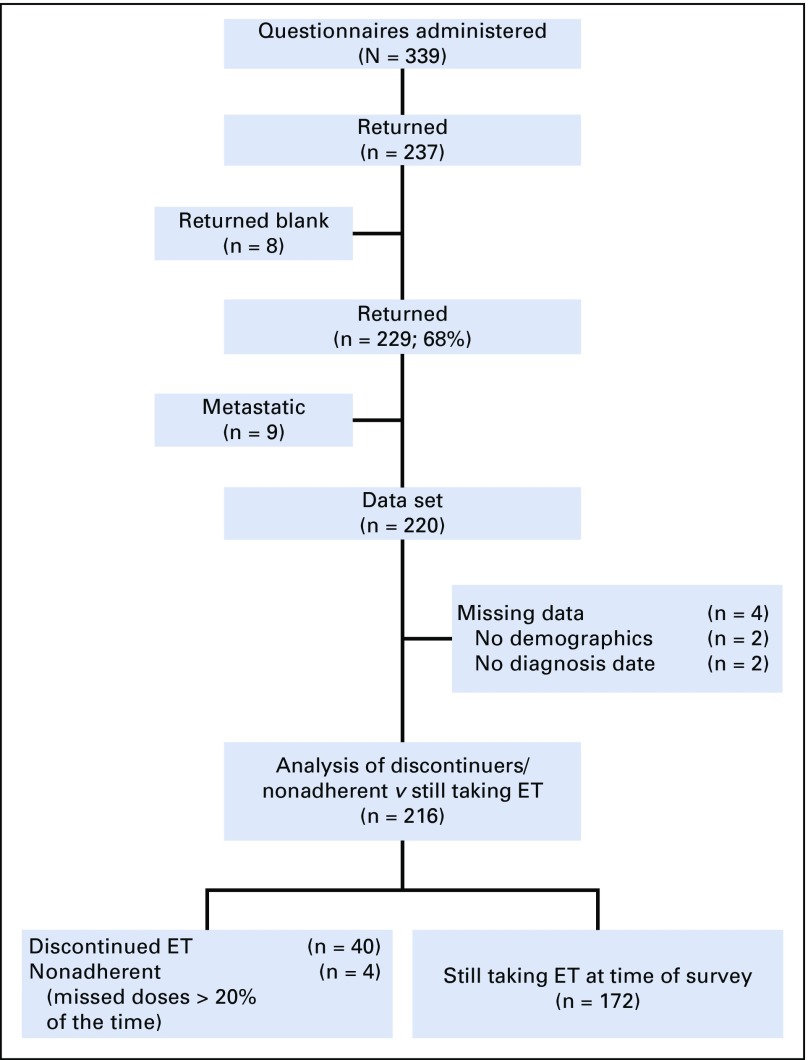
Response rate. ET, endocrine treatment.

**FIG 2.**
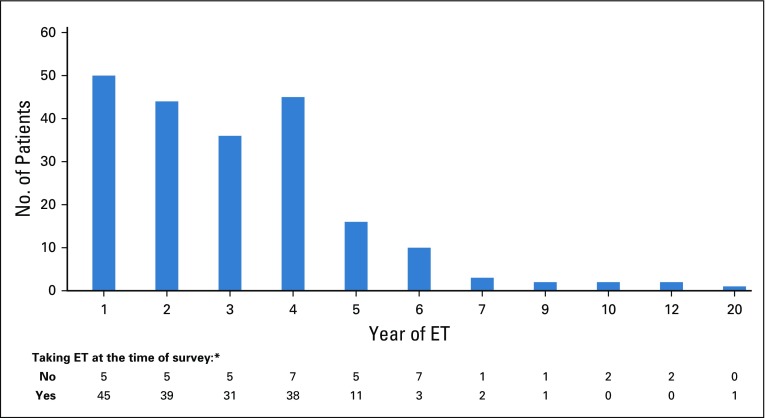
Distribution of all participants at time they completed questionnaire. (*) Excludes the four participants who reported missing ≥ 20% of endocrine treatment (ET) doses.

From February to April 2013, a total of 339 questionnaires were distributed to patients who had been prescribed adjuvant ET for hormone receptor–positive, HER2-negative breast cancer. Of the 237 that were returned, eight were blank, leaving 229 (return rate, 68%). Complete data were available for 216 participants (Fig 1). Sixty-five percent of the sample self-identified as white. Seventy-seven percent of the sample had either attended college, received a college degree, or received an advanced degree, whereas 23% reported having received a high school diploma, general educational development, or less education (Table 1). A majority of participants were in the first five years of ET when they completed the survey; 10 patients were in year 6 of their ET, three patients were in year 7, two were in year 9, two were in year 10, two were in year 12, and one was in year 20 (Fig 2). The duration of adherence of the sample to ET ranged from never initiated to 20 years (median, 3.00 years; standard deviation [SD], 2.04 years). We included participants who were beyond the initial 5 years of ET, because patients at MDA were asked to extend ET for at least 10 years starting in 2005.^11,12^

**TABLE 1.**
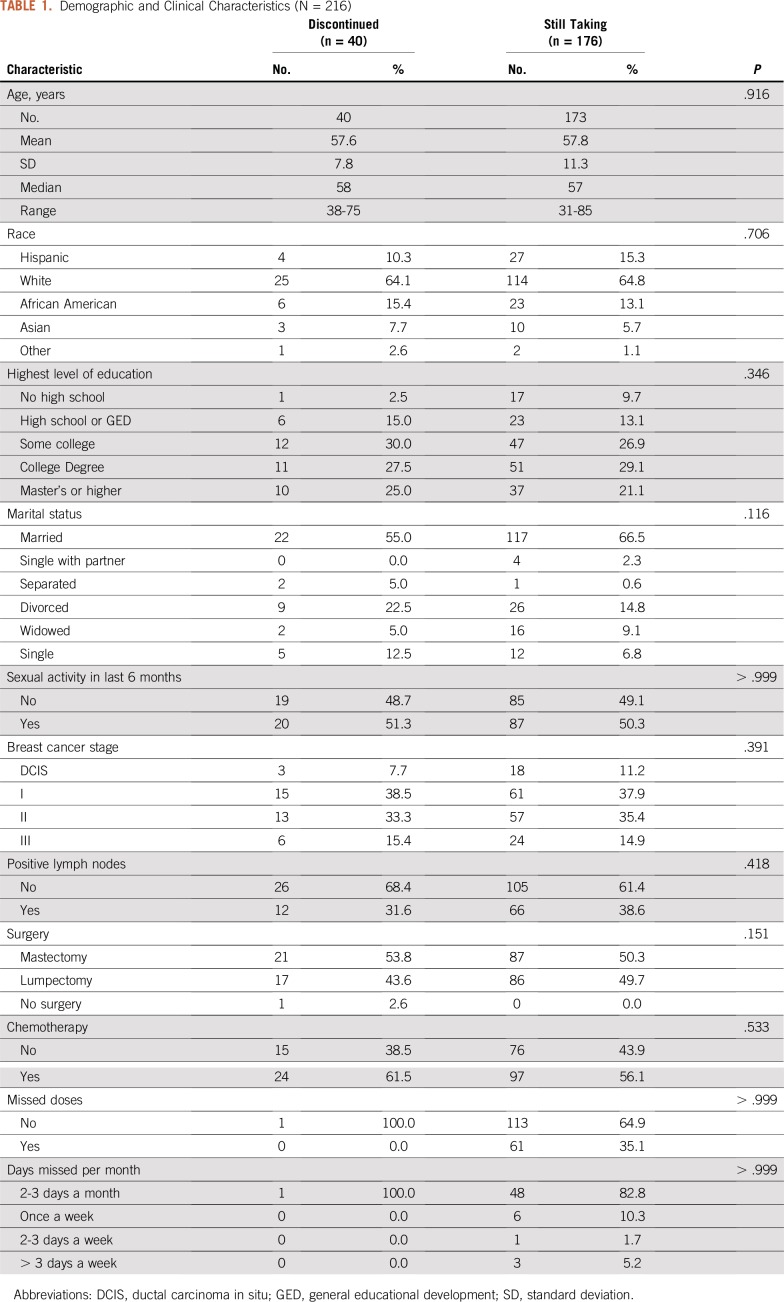
Demographic and Clinical Characteristics (N = 216)

### Number of Nonadherent and Discontinued Patients

**FIG 3.**
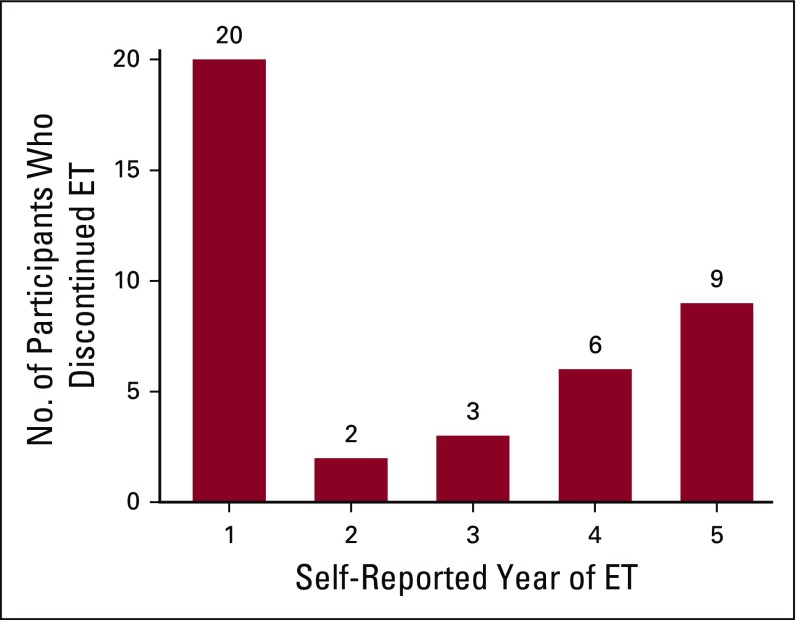
Self-reported year of endocrine treatment (ET) discontinuation (n = 40).

True overall prevalence of nonadherence could not be calculated, because this was not a prospective cohort study. Assuming that participants who had reported discontinuation in early years remained discontinued in subsequent years, 40 of 216 participants discontinued their ET, and all 40 had discontinued by year 5 (Fig 2). Among the 40 discontinued patients, discontinuation rates peaked during year 1 of ET (50% of discontinued patients reported compliance with ET for < 1 year) and in years 4 and 5 (37.5% of discontinued patients; Fig 3). An additional four patients (1.8%) answered that they were still taking ET but were missing doses > 2 days a week.

There were a total of 20 patients who were beyond their fifth year of surveillance when they answered the anonymous questionnaire. Of these 20, seven patients reported that they had elected to extend their ET beyond the standard 5 years and were still taking their daily pill. The remaining 13 patients (65%) reported that they had discontinued taking their ET within the first 5 years, and therefore, the decision to extend ET beyond 5 years was not relevant.

### Demographic and Clinical Factors Unassociated With Nonadherence

None of the demographic or clinical characteristics were significantly correlated with dichotomized adherence status (Table 1) and therefore not included as control variables in the iterative logistic regression analyses. When the 13 ET adverse effects/reasons for discontinuation were entered into a single logistic regression equation for nonadherence, we found extensive and significant Spearman correlation coefficients linking a large fraction of the 13 reasons, especially among adherent participants and among those who had become nonadherent during years 4 to 6. To address this collinearity,^16^ we then conducted separate logistic regression analyses for each of the 13 ET reasons for nonadherence to isolate the impact of each reason and circumvent collinearity.

### Simulated Time Dependence of Reasons for Nonadherence

The results from the iterative logistic regression analyses over windowed time periods showed that all 13 ET adverse effects and reasons were significantly related to nonadherence at some point during the first 6 years of ET (tests used two-sided *P* value distributions; Table 2). However, with the exception of bone and joint pain (*P* < .001 to *P* = .04), the reasons were inconsistently associated with adherence status over time.

**TABLE 2.**
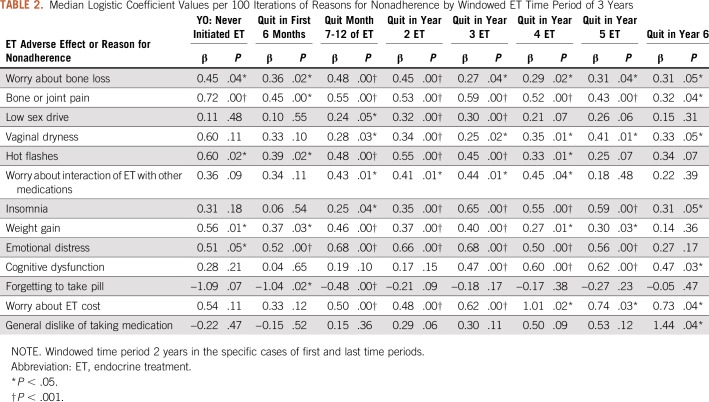
Median Logistic Coefficient Values per 100 Iterations of Reasons for Nonadherence by Windowed ET Time Period of 3 Years

**FIG 4.**
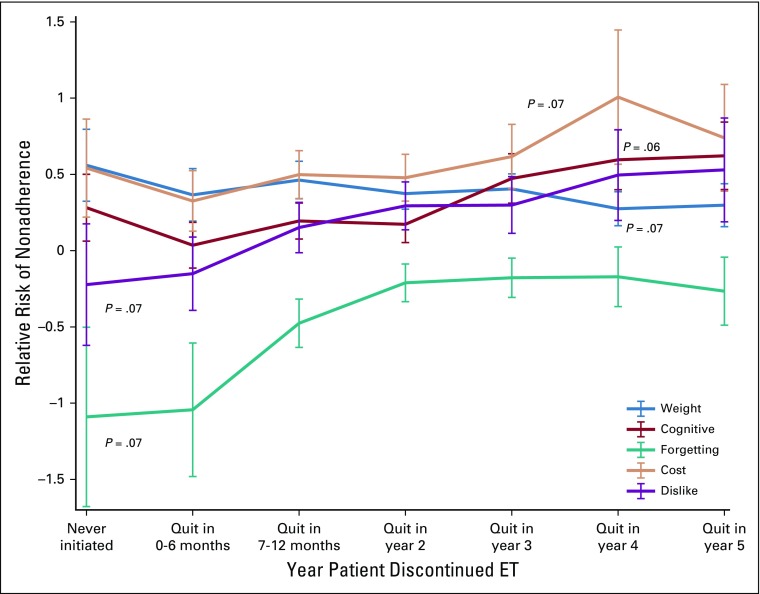
Nonrandom change in impact over time on nonadherence to endocrine treatment (ET).

The change in impact of reasons over time was illustrated graphically by expressing the median value for each logistic regression coefficient as an OR (OR = *e* raised to the power of the coefficient, with an OR of 1.0 equal to a coefficient of zero), plotted as a function of each windowed time period. When testing the null hypothesis that these changes in coefficients over time were random, none of the reason sequences achieved significance of *P* < .05. However, five reasons approached significance, with *P* < .10, including weight gain (*P* = .07), cognitive dysfunction (*P* = .06), worry about ET cost (*P* = .07), general dislike of taking medication (*P* = .07), and forgetting to take medication (*P* = .07), meaning that these reasons displayed a ≤ 7% chance of being a random sequence (Fig 4). Conversely, insomnia, bone and joint pain, hot flashes, worry about bone loss, vaginal dryness, emotional distress, decreased sex drive, and worry about ET interaction with other prescribed medications did not show a strong trend across time (*P* = 0.29 to 1.00) and thus exercised relatively stable influences on nonadherence during the first 6 years of ET.

Across all time periods in the ET trajectory, worry about ET cost had the highest overall median OR (1.79; Std Dev= 0.42), and became increasingly important after year 3. Emotional distress had the second highest overall median OR (1.72; SD, 0.22), and its influence on nonadherence peaked after the second year of ET. Bone and joint pain had the third highest overall median OR (1.69; SD, 0.20), and its association with nonadherence was especially strong at the outset of ET, decreasing slightly and remaining relatively constant thereafter.

## DISCUSSION

Assuming that participants who reported discontinuation during any given year of ET remained so during subsequent years, 40 participants of the cohort of 216 discontinued ET during the first 5 years. For the 20 participants who were beyond their fifth year of ET (n = 20), a majority (65%) did not extend their ET beyond the fifth year. We expected that our sample of participants would have higher adherence to ET, because MDA tends to attract patients with access to high-quality insurance, and found this to be the case, with our overall rate of nonadherence (20.3%) being lower than the range reported in large population-based studies (ie, 40% to 60%).^2-4,17^ Our sample was restricted to patients who were still returning to MDA for surveillance and may have been biased toward inclusion of longer-term survivors with more economic resources. Also, patients with a complicated course of adjuvant treatment and recovery may have been more likely to continue surveillance visits at MDA rather than transitioning back to a primary care provider and thus may have received more attention from the oncology team regarding ET adherence. This aspect remains to be studied.

In contrast to previously reported literature, age, stage, treatment, and race were not related to discontinuation status. This may have resulted from the lack of variability in these factors within the patient population at MDA. Patients who reported forgetting to take their ET at least some of the time were not more likely to have discontinued ET. Thus, forgetting might be interpreted as persistent effort to adhere to the medication, rather than a lack of commitment.

A critical gap in the adherence intervention literature, especially as it pertains to chronic self-administration of daily oral therapy, is the lack of understanding as to how and why patients’ adherence changes over time. We used iterative bootstrapping and subsampling methods as a novel way to address a common problem in the field (ie, lack of prospective, repeated patient-reported data). Given this limitation, analytic simulations resulted in several interesting findings that could not have otherwise been ascertained with traditional statistical analyses. Although these effects did not achieve statistical significance in these limited initial data, results of our regression analysis suggest that worry about cost, a general dislike of pills, and complaints of cognitive dysfunction may be associated with nonadherence during the latter years rather than the early years of ET (*P* < .10). In contrast, bone and joint pain was consistently associated with nonadherence across all time periods.

Worry about the cost of ET, which had the highest median value of association with nonadherence, has not been examined as extensively as physical adverse effects of ET.^6^ It is interesting that concern about the cost of ET had the strongest association with nonadherence in later years within a sample where a majority of patients have access to high-quality medical insurance plans that allow continued care at a tertiary cancer center. This finding suggests that worry about cost might be even more salient in community practice settings.

Only seven of 20 patients elected to continue ET beyond the standard 5 years, despite being encouraged to do so by their medical oncologists. Although our cohort of patients who were beyond the fifth year of ET was small, this finding suggests that adherence to ET beyond 5 years may be problematic within the context of emerging evidence supporting the added benefit of extending ET to ≥ 10 years.^11,18^ It should be emphasized that the number of participants was limited in this first study and that continued analysis must be performed as new data are acquired.

Our study design assessed patients at a single time point, which prevented us from ascertaining true 5-year nonadherence rates. For example, it cannot be assumed that all participants who were within the first few years of ET and who were adherent at the time of the survey would have remained adherent by the end of the fifth year of ET. Another limitation was that the small sample size of discontinued and nonadherent (missing > 20% doses) patients precluded the ability to control for demographic, tumor, or treatment variables within each separate regression analysis. Finally, the generalizability of our study is limited, because the sample came from a single clinic.

In conclusion, we used iterative bootstrapping and subsampling methods as a novel way to analyze prospective, repeated patient-reported data, leading to several interesting findings that could not have otherwise been ascertained with traditional statistical analyses. These results show that the influence of some reasons change over time, whereas other reasons exact a stable influence on discontinuation. Although some reasons for discontinuation exerted a steady influence over the 6-year ET trajectory (eg, bone and joint pain), other reasons, such as cost, cognitive complaints, and general dislike of pills, became more important in the later years of ET. These data illustrate the utility of simulation modeling to describe the changing impact of reasons for nonadherence during the course of treatment. Future studies detailing the trajectories of behavioral changes leading to discontinuation of ET may provide potentially useful targets for intervention to prevent discontinuation of ET.

## Data Availability

The following represents disclosure information provided by authors of this manuscript. All relationships are considered compensated. Relationships are self-held unless noted. I = Immediate Family Member, Inst = My Institution. Relationships may not relate to the subject matter of this manuscript. For more information about ASCO's conflict of interest policy, please refer to www.asco.org/rwc or ascopubs.org/jco/site/ifc. **Stock and Other Ownership Interests:** Pfizer **Honoraria:** Novartis, Pfizer (I) **Research Funding:** Oncothyreon (Inst), Pfizer (Inst), Novartis (Inst), Genentech (Inst), Takeda Pharmaceuticals (Inst), Bayer HealthCare Pharmaceuticals (Inst), EMD Serono (Inst) **Travel, Accommodations, Expenses:** Novartis, Pfizer (I) **Stock and Other Ownership Interests:** ISIS Pharmaceuticals, Nuvera Biosciences, Delphi Diagnostics **Consulting or Advisory Role:** Merck Sharp & Dohme **Patents, Royalties, Other Intellectual Property:** Intellectual property **Travel, Accommodations, Expenses:** Luminex, Merck Sharp & Dohme No other potential conflicts of interest were reported.
